# Data-driven exploration of occupations and lung cancer risks using regularized regression models

**DOI:** 10.1093/ije/dyag183

**Published:** 2026-10-23

**Authors:** Calvin Ge, Jia Nie, Lützen Portengen, Ann Olsson, Joachim Schüz, Wolfgang Ahrens, Christine Barul, Thomas Behrens, Thomas Brüning, Neil E Caporaso, Dario Consonni, Paul A Demers, Eleonora Fabianova, Guillermo Fernández-Tardón, John K Field, Francesco Forastiere, Lenka Foretova, Per Gustavsson, Vladimir Janout, Karl-Heinz Jöckel, Stefan Karrasch, Benjamin Kendzia, Hans Kromhout, Maria Teresa Landi, Jolanta Lissowska, Dana Mates, John R McLaughlin, Franco Merletti, Enrica Migliore, Tamás Pándics, Loredana Radoi, Lorenzo Richiardi, Miriam Schejbalova, Jack Siemiatycki, Beata Świątkowska, Heinz-Erich Wichmann, David Zaridze, Kurt Straif, Susan Peters, Roel Vermeulen

**Affiliations:** Institute for Risk Assessment Sciences, Utrecht University, Utrecht, The Netherlands; Risk Analysis for Prevention, Innovation & Development, Unit Health and Work, Netherlands Organization for Applied Scientific Research TNO, Utrecht, The Netherlands; Institute for Risk Assessment Sciences, Utrecht University, Utrecht, The Netherlands; Institute for Risk Assessment Sciences, Utrecht University, Utrecht, The Netherlands; The Environment and Lifestyle Epidemiology Branch, International Agency for Research on Cancer (IARC/WHO), Lyon, France; The Environment and Lifestyle Epidemiology Branch, International Agency for Research on Cancer (IARC/WHO), Lyon, France; Department of Epidemiological Methods and Etiological Research, Leibniz Institute for Prevention Research and Epidemiology—BIPS, Bremen, Germany; Faculty of Mathematics and Computer Science, Institute of Statistics, University of Bremen, Bremen, Germany; Irset (Institut de recherche en santé, environnement et travail) - UMR_S 1085, Univ Rennes, Inserm, EHESP, Pointe-à-Pitre, France; Institute for Prevention and Occupational Medicine of the German Social Accident Insurance, Institute of the Ruhr University (IPA), Bochum, Germany; Institute for Prevention and Occupational Medicine of the German Social Accident Insurance, Institute of the Ruhr University (IPA), Bochum, Germany; Division of Cancer Epidemiology and Genetics, National Cancer Institute, NIH, Bethesda, MD, United States; Epidemiology Unit, Fondazione IRCCS Ca’ Granda Ospedale Maggiore Policlinico, Milan, Italy; Occupational Cancer Research Centre, Ontario Health, Toronto, ON, Canada; Department of health Risk Assessment, Regional Authority of Public Health, Banská Bystrica, Slovakia; Faculty of Health, Catholic University, Ružomberok, Slovakia; Health Research Institute of Asturias, University of Oviedo, ISPA and CIBERESP, Oviedo, Spain; Roy Castle Lung Cancer Research Programme, Department of Molecular and Clinical Cancer Medicine, University of Liverpool, Liverpool, United Kingdom; Department of Epidemiology, ASL Roma E, Rome, Italy; Department of Cancer Epidemiology and Genetics, Masaryk Memorial Cancer Institute, Brno, Czechia; Institute of Environmental Medicine, Karolinska Institutet, Stockholm, Sweden; Faculty of Health Sciences, Palacky University, Olomouc, Czechia; Institute for Medical Informatics, Biometry and Epidemiology (IMIBE), University Hospital Essen, Essen, Germany; Institute and Clinic for Occupational, Social and Environmental Medicine, LMU University Hospital, LMU Munich, Munich, Germany; Comprehensive Pneumology Center Munich (CPC-M), German Center for Lung Research (DZL), Munich, Germany; Institute of Epidemiology, Helmholtz Zentrum München—German Research Center for Environmental Health, Neuherberg, Germany; Institute for Prevention and Occupational Medicine of the German Social Accident Insurance, Institute of the Ruhr University (IPA), Bochum, Germany; Institute for Risk Assessment Sciences, Utrecht University, Utrecht, The Netherlands; Division of Cancer Epidemiology and Genetics, National Cancer Institute, NIH, Bethesda, MD, United States; Department of Cancer Epidemiology and Prevention, Epidemiology Unit, M. Sklodowska-Curie National Research Institute of Oncology, Warsaw, Poland; Environmental Health Department, National Institute of Public Health, Bucharest, Romania; Dalla Lana School of Public Health, University of Toronto, Toronto, ON, Canada; Department of Medical Sciences, Cancer Epidemiology Unit, University of Turin, Turin, Italy; Department of Medical Sciences, Cancer Epidemiology Unit, University of Turin, Turin, Italy; Public Health Laboratory Department, National Public Health Center, Budapest, Hungary; Team Exposome and Heredity, Center for Research in Epidemiology and Population Health (CESP), U1018 Inserm, University Paris-Saclay, University Paris Cité, Villejuif, France; Department of Medical Sciences, Cancer Epidemiology Unit, University of Turin, Turin, Italy; First Faculty of Medicine, Institute of Hygiene and Epidemiology, Charles University, Prague, Czechia; Department of Social and Preventive Medicine, University of Montreal, Montreal, QC, Canada; Department of Environmental Epidemiology, The Nofer Institute of Occupational Medicine, Lodz, Poland; Institute of Epidemiology, Helmholtz Zentrum München—German Research Center for Environmental Health, Neuherberg, Germany; Institut für Medizinische Informatik Biometrie Epidemiologie, Ludwig Maximilians University, Munich, Germany; Department of Cancer Epidemiology and Prevention, N.N. Blokhin National Research Centre of oncology, Moscow, Russia; Environment and Health over the Lifecourse, ISGlobal, Barcelona, Spain; Department of Biology, Boston College, MA, United States; Institute for Risk Assessment Sciences, Utrecht University, Utrecht, The Netherlands; Institute for Risk Assessment Sciences, Utrecht University, Utrecht, The Netherlands

**Keywords:** occupation, lung cancer, Bayesian hierarchical model, LASSO regression

## Abstract

**Background:**

Occupational epidemiological analyses typically focus on *a priori* hypotheses relating disease to specific exposures or occupations. We used regularized regression models to agnostically identify occupations associated with lung cancer while accounting for known lung carcinogens.

**Methods:**

We pooled data from 14 European and Canadian lung cancer case–control studies. The main analysis included 13 587 cases and 16 410 controls for men and 3199 cases and 4408 controls for women. Associations between ever-employment in various occupations and lung cancer risks were evaluated using two selection approaches: a least absolute shrinkage and selection operator regression and a two-stage Bayesian hierarchical horseshoe model accounting for age, study, smoking, and occupational exposure to known lung carcinogens. Unconditional logistic regression models were used to estimate odds ratios (ORs) and 95% confidence intervals (CIs) for selected occupations.

**Results:**

For men, occupations consistently identified as positively associated with lung cancer included miners (OR = 1.28, 95% CI: 1.06–1.54) and bricklayers (OR = 1.30, 95% CI: 1.12–1.52), while secondary education teachers (OR = 0.37, 95% CI: 0.22–0.60), production managers (OR = 0.66, 95% CI: 0.54–0.81), and government executive officials (OR = 0.72, 95% CI: 0.62–0.83) were negatively associated. For women, secondary language and literature teachers (OR = 0.39, 95% CI: 0.20–0.72) and government executive officials (OR = 0.63, 95% CI: 0.48–0.81) showed negative associations.

**Conclusion:**

Two agnostic, data-driven models identified several occupations associated with both increased and decreased lung cancer risks. These associations persisted after accounting for known exposures and covariates, highlighting the need to investigate unidentified occupational factors.

Key Messages

- Data-driven models were used to evaluate associations between occupations and lung cancer while accounting for several known lung carcinogenic exposures.
- Lung cancer associations for specific occupations, such as miners and construction bricklayers, were identified and quantified while accounting for smoking and occupational exposures to multiple known lung carcinogens.
- We demonstrate the utility of agnostic modelling approaches in identifying lung cancer associations with individual occupation and highlight potential directions for further research to elucidate the mechanisms underlying the observed associations.

## Background

Occupational epidemiological studies have helped elucidate the aetiology of external exposures and health outcomes. Many exposure–disease associations were first demonstrated in working populations; examples include asbestos and asbestosis [1] and diesel motor exhaust and lung cancer [2]. Recent studies in occupational epidemiology typically focus on the identification and quantification of associations between health outcomes and *a priori* hypothesized exposure or occupation. These hypothesis-driven studies have produced substantial insight into exposure-response relations over the last several decades.

In addition, large datasets from modern population-based studies present an opportunity for an agnostic, data-driven approach that is complementary to hypothesis-driven approaches. Data-driven exploratory approaches may highlight knowledge gaps that were missed in hypothesis-driven research and help formulate new research hypothesis. Data-driven methods and research have been applied in multiple other research areas, including environmental health and cancer-metabolomics studies [3, 4]. However, the application of such methods has been limited in occupational epidemiological studies, particularly for exploring disease risks associated with employment in different occupations.

In a pooled, population-based lung cancer study of over 16 000 cases and 20 000 controls, we applied two exploratory, data-driven modelling approaches to occupation-level associations with lung cancer, accounting for multiple known lung carcinogens. Our first approach utilized least absolute shrinkage and selection operator (LASSO) penalized regressions, and the second Bayesian hierarchical models.

## Methods

### Study population

The study is based on the pooled lung cancer case–control study, SYNERGY (http://synergy.iarc.fr). This study is described in detail elsewhere [5]. Briefly, 16 901 cases and 20 965 controls were pooled from 14 hospital- and population-based studies in 13 European countries and Canada. Information available for study subjects includes age, sex, cigarette use habits including amount and cessation, full occupational history, and quantitative assessment of occupational exposure to six lung carcinogens: asbestos, diesel engine exhaust, hexavalent chromium, nickel, polycyclic aromatic hydrocarbons, and respirable crystalline silica. Exposure assessments to these carcinogens were assigned by the SYN-JEM and DEE-JEM [6, 7]. Cumulative exposure to benzene was also quantified for participants whose job records were from 1945 to 2009 [8]. Participants without employment history (*n* = 262) were excluded.

All jobs reported by SYNERGY participants were coded in the International Standard Classification of Occupations, version 1968 (ISCO-68) [9]. ISCO-68 is a hierarchical occupation classification system in which the highest detail is presented in five-digit groupings. Broader groupings of occupations occur at the three-, two-, and one-digit levels. For example, the three-digit group ‘9-51 bricklayers, stonemasons and tile setters’ includes more specific five-digit occupations such as ‘9-51.20 bricklayer (construction)’, ‘9-51.30 firebrick layer’, ‘9-51.40 stonemason’, etc. In this manuscript, unless otherwise specified, subsequent mentions of ‘occupation’ refer to the five-digit ISCO-68 occupation categories, and mentions of ‘job’ refer to job records reported by study participants.

Participants’ jobs with sufficient information to be coded to a three-digit level or higher were included. We excluded three-digit level occupations for which ever-employment involved fewer than five subjects or ever-employment involved only case or control subjects. Thus, for men (*n* = 29 997), ever or never employment in the 274 three-digit occupations and 1356 sub-level five-digit occupations were included in the analyses. For women (*n* = 7607), ever or never employment in 212 three-digit occupations and 598 sub-level five-digit occupations were included in the analyses.

### LASSO with stability selection

To evaluate potential associations between lung cancer and ever-employment in various ISCO-68 occupations, we implemented logistic regression analyses using LASSO-penalized regression combined with complementary pairs stability selection using the R package *stabs* [10]. The procedure is detailed elsewhere [11, 12]. Briefly, the dataset was randomly divided into two equal-sized sub-samples, and LASSO regressions were performed in both subsets. In each analysis, the data subsetting and LASSO regressions were run in each; this was repeated 50 times, giving 100 individual LASSO regressions. Based on the specified per-family error rate (PFER) and selection frequency (cutoff), each round of LASSO regression selects a certain number of variables, and the variables with selection rates beyond the specified cutoff become the final ‘stable selection’.

Specifically, for the current study, the base logistic regression model for LASSO with stability selection consisted of all included five-digit ISCO-68 occupations (ever/never) and the following covariates: age group (<45, 45–49, 50–54, 55–59, 60–64, 65–69, 70–74, and >74 years), study centre, smoking (log(cigarette pack-years + 1)), and smoking cessation (current smokers; >0–7; 8–15; 16–25; >25 years; never smokers). The full LASSO model was identical to the base model plus additional covariates representing six individual quantitative cumulative exposure variables for the following occupational carcinogens: asbestos, diesel engine exhaust, hexavalent chromium, nickel, polycyclic aromatic hydrocarbons, and respirable crystalline silica. Both the base and full LASSO models were performed stratified by sex. A cut-off rate of 0.75 was used with various PFER values (10, 5, 2, 1) to explore models with different tolerances for potential false-positive findings. Given the exploratory aim of our work, we present results using PFER = 10 as our main model.

### Bayesian hierarchical horseshoe models

To account for the nested, inter-related structure of the occupation classification system, we implemented a Bayesian hierarchical horseshoe model. For computational reasons, this model was developed as a two-stage model.

The base and full first-level models (M1) consisted of unconditional logistic regressions with identical covariates and sex stratification as the base and full LASSO models, respectively. The M1 effect estimates and their standard errors were then used to construct second-level hierarchical models (M2) in the Bayesian framework with the following structure:


$$\begin{array}{c} {\beta \mathrm{obs}}_{i}|{\beta \exp}_{i},\;\varepsilon_{i}^{2}∼N({\beta \exp}_{i},\;\varepsilon_{i}^{2})\\ \begin{array}{c} {\beta \exp}_{i}∼\mathrm{HS}(b_{g_{i}},\delta_{\mathrm{isco}5})\\ b_{j}∼\mathrm{HS}(0,\delta_{\mathrm{isco}3}) \end{array} \end{array}$$

where *i* indexes the ISCO-68 five-digit occupations, *j* the ISCO-68 three-digit occupation groups, and *g_i_* is the ISCO-68 three-digit occupation group to which the *i*th ISCO-68 five-digit occupation belongs. ${\beta \mathrm{obs}}_{i}$ is the coefficient estimate for the *i*th ISCO-68 five-digit occupation from M1, and $\varepsilon_{i}$ is the corresponding standard error. Regularization of coefficients in the Bayesian hierarchical model was parameterized with horseshoe priors as implemented by Piironen and Vehtari [13]. We use the notation HS(b,δ) to indicate the modified horseshoe prior, where b is an offset and a conventional horseshoe prior is assigned to deviations from this offset with parameters δ. We used the brms default prior settings: student-t priors with df = 1 for the local shrinkage parameters, df_global = 1 for the global shrinkage parameter, and df_slab = 4 and scale_slab = 2 for the regularization parameter. The scale of the student-t prior for the global shrinkage parameter (scale_global) was selected assuming equal odds for the expected number of zero and nonzero coefficients, divided by the number of observations, separately for the five-digit ISCO-68 occupations and the three-digit ISCO-68 occupation groups. Occupations with an estimated increased or decreased risk of at least 20% and a 90% credible interval not overlapping 1.0 were considered “selected”.

### Combined inference

To illustrate how selection results from the LASSO and Bayesian hierarchical approaches may be combined, we used unconditional logistic regression models to estimate the risk of lung cancer with occupations selected by both approaches. The odds ratios (ORs) and 95% CIs for ever employment in five-digit occupation-specific risk were calculated, adjusting for identical covariates as the full LASSO models. To further explore the risk among the selected occupations after accounting for potential occupational carcinogens, we calculated *E*-values [14] based on the occupation-specific estimates. The *E*-value quantifies how strong an unmeasured confounder association would need to be to explain an observed association [15]; larger *E*-values indicate stronger required confounding.

Subgroup analyses were conducted according to smoking status, and interactions with ever-employment in the selected occupations were tested. Sensitivity analyses were performed by further adjusting for cumulative occupational benzene exposure in participants whose job records were within the period of 1945–2009. These analyses were done because we recently found a consistent association between benzene exposure and lung cancer in this population [8]. Further sensitivity analyses were conducted using an alternative definition of ever employment for jobs with 5 or more years of employment.

All statistical analyses were performed in R, version 4.2.3 [16].

## Results

The main analysis included 13 587 cases and 16 410 controls for men and 3199 cases and 4408 controls for women. A total of 25 and 8 ISCO-68 occupations were selected by the full LASSO models for men and women, respectively (Tables 1 and 2). Occupations selected by the LASSO models for men versus women were mostly non-overlapping, except ‘first-level education teachers’ (1-33.20) and ‘government executive official’ (3-10.10), for which a negative association with lung cancer was found for both sexes. For men, occupations with the largest association magnitudes in the full LASSO model were ‘carpenters’ (9-54.10) and ‘dockers’ (9-71.20) in the positive direction and ‘mechanical engineer’ (0-24.10) and ‘other secondary education teachers’ (1-32.90) in the negative direction. The corresponding occupations for women were ‘other production and related workers’ (9-49.90) and ‘waiter general’ (5-32.10) for the positive direction and ‘second-level languages and literature teacher’ (1-32.15) and ‘government executive official’ (3-10.10) for the negative direction.

**Table 1:** Occupations ever-employed by men selected using least absolute shrinkage and selection operator (LASSO) penalized regression models (*N* = 29 997).^a^

ISCO-68	Job title	Controls, *n* (%)	Cases, *n* (%)	Lung cancer association directionality^b^	
				Base model	Full model
0-24.10	Mechanical engineer (general)	165 (1.0%)	50 (0.4%)	−0.613	−0.567
0-32.10	Draughtsman, general	154 (0.9%)	82 (0.6%)	−0.260	−0.227
1-31.90	Other university and higher education teachers	89 (0.5%)	23 (0.2%)	−0.526	−0.480
1-32.90	Other secondary education teachers	97 (0.6%)	24 (0.2%)	−0.602	−0.562
1-33.20	First-level education teachers	150 (0.9%)	53 (0.4%)	−0.325	−0.287
2-12.10	Production manager (except farm)	358 (2.2%)	182 (1.4%)	−0.377	−0.348
2-19.40	Administration manager	188 (1.2%)	81 (0.6%)	−0.226	−0.202
3-10.10	Government executive official	717 (4.6%)	431 (3.3%)	−0.305	−0.271
3-31.10	Bookkeeper (general)	211 (1.3%)	102 (0.8%)	−0.295	−0.272
3-93.10	Office clerk (general)	824 (5.3%)	508 (3.9%)	−0.186	−0.151
4-31.30	Technical service adviser	107 (0.7%)	41 (0.3%)	−0.500	−0.471
4-32.20	Commercial traveller	483 (3.0%)	345 (2.6%)	−0.208	−0.186
5-82.20	Policemen	292 (1.8%)	240 (1.8%)	−0.174	−0.144
7-11.05	Miner (general)	274 (1.7%)	454 (3.5%)	+0.321	+0.198
8-34.20	Lathe operator	356 (2.2%)	307 (2.3%)	−0.128	−0.146
8-41.05	Machinery fitter (general)	380 (2.4%)	397 (3.0%)	+0.120	0
8-43.20	Automobile mechanic	490 (3.1%)	503 (3.8%)	+0.076	0
8-49.10	Machinery mechanic (general)	312 (1.9%)	388 (2.9%)	+0.215	+0.199
8-72.10	Gas and electric welder (general)	241 (1.5%)	340 (2.6%)	+0.207	+0.184
9-31.20	Building painter	265 (1.6%)	351 (2.7%)	+0.235	+0.251
9-51.20	Bricklayer (construction)	412 (2.6%)	618 (4.8%)	+0.291	+0.227
9-54.10	Carpenter (general)	107 (0.7%)	171 (1.3%)	+0.436	+0.458
9-69.30	Boiler fireman	74 (0.5%)	133 (1.0%)	+0.301	0
9-71.20	Docker	71 (0.4%)	109 (0.8%)	+0.397	+0.394
9-74.20	Excavating machine operator	75 (0.5%)	119 (0.9%)	+0.357	+0.347
9-85.60	Lorry and van driver (long-distance transport)	478 (3.0%)	547 (4.2%)	+0.077	+0.103
9-99.10	Labourer	1200 (7.9%)	1416 (11.6%)	+0.119	+0.087
	Armed forces^c^	834 (5.4%)	889 (7.0%)	+0.103	+0.102

a LASSO regression with stability selection was set to a cut-off rate of 0.75 (which means any job with a selection probability higher than 0.75 will be selected) and PFER of 10 (maximum tolerated number of Type I errors among selections were 10). The base model used all five-digit ISCO-68 jobs (*n* = 1356) that ever employed by men and adjusted for the following covariates: age group (<45, 45–49, 50–54, 55–59, 60–64, 65–69, 70–74, >74 years), study centre, smoking (log(cigarette pack-years + 1)), smoking cessation (current smokers; >0–7; 8–15; 16–25; >25 years; never smokers). The full model further adjusted six individual quantitative cumulative exposure variables for the following occupational carcinogens: asbestos, diesel engine exhaust, hexavalent chromium, nickel, polycyclic aromatic hydrocarbons, and respirable crystalline silica.

b Selected jobs indicated by ‘+’ or ‘−’ and the shrunken coefficient per job title, representing ‘positive’ or ‘negative’ associations with lung cancer risk.

c No ISCO-68 code is available for armed forces.

**Table 2:** Occupations ever employed by women selected using least absolute shrinkage and selection operator (LASSO) penalized regression models (*N* = 7607).^a^

ISCO-68	Job title	Controls, *n* (%)	Cases, *n* (%)	Lung cancer association directionality^b^	
				Base model	Full model
1-32.15	languages and literature teacher (second level)	57 (1.3%)	16 (0.5%)	−0.218	−0.138
1-33.20	first level education teachers	156 (3.7%)	61 (1.9%)	−0.121	−0.103
3-00.10	clerical supervisor (general)	57 (1.3%)	21 (0.7%)	−0.009	0
3-10.10	government executive official	206 (4.9%)	140 (4.6%)	−0.316	−0.310
3-39.40	finance clerk	50 (1.1%)	44 (1.4%)	+0.101	+0.021
3-94.10	receptionist (general)	95 (2.2%)	41 (1.3%)	−0.120	−0.068
5-32.10	waiter general	151 (3.5%)	197 (6.6%)	+0.130	+0.228
5-52.20	charworkers	348 (8.6%)	339 (11.9%)	+0.119	+0.112
9-49.90	other production and related workers nec	60 (1.4%)	76 (2.4%)	+0.312	+0.263

a LASSO regression with stability selection was set a cut-off rate of 0.75 (which means any job with a selection probability higher than 0.75 will be selected) and PFER of 10 (maximum tolerated number of Type I errors among selections were 10). The base model used all five-digit ISCO-68 jobs (*n* = 586) that ever employed by women and adjusted for the following covariates: age group (<45, 45–49, 50–54, 55–59, 60–64, 65–69, 70–74, >74 years), study centre, smoking (log(cigarette pack-years + 1)), smoking cessation (current smokers; >0–7; 8–15; 16–25; >25 years; never smokers). Full model further adjusted six individual quantitative cumulative exposure variables for the following occupational carcinogens: asbestos, diesel engine exhaust, hexavalent chromium, nickel, polycyclic aromatic hydrocarbons, and respirable crystalline silica.

b Selected jobs indicated by ‘+’ or ‘−’ and the shrunken coefficient per job title, representing ‘positive’ or ‘negative’ associations with lung cancer risk.

A total of 23 and 11 ISCO-68 occupations were selected by the full Bayesian hierarchical models for men and women, respectively (Tables 3 and 4). For both sexes, many of the five-digit occupations selected were within the three-digit unit group ‘secondary education teacher (1-32)’, where a negative association with lung cancer was observed. Additionally, for men, miners (7-11.05) as well as occupations within the ‘bricklayers, stonemasons and tile setters (9-51)’ three-digit unit group were identified with a positive association with lung cancer.

**Table 3:** Occupations ever employed by men selected using Bayesian hierarchical penalization models (*N* = 29 997).^a^

3-digit ISCO	5-digit ISCO	Job titles	Controls, *n* (%)	Cases, *n* (%)	ORs (90%CrIs)	
					Base model	Full model
0-24	0-24.10	Mechanical engineer (general)	165 (1.0%)	50 (0.4%)	**0.55 (0.36**–**0.98)**	0.57 (0.37–1.00)
0-83	0-83.00	Systems analysts	11 (0.1%)	1 (0.0%)	0.57 (0.22–1.03)	0.56 (0.22–1.03)
	0-83.10	Systems analyst	63 (0.4%)	11 (0.1%)	0.54 (0.22–1.02)	0.54 (0.22–1.02)
1-21	1-21.10	Lawyer	50 (0.3%)	21 (0.2%)	0.78 (0.40–1.04)	0.80 (0.41–1.04)
1-31	1-31.55	Teacher in education (third level)	8 (0.0%)	7 (0.1%)	2.21 (0.76–17.12)	2.14 (0.77–17.04)
1-32	1-32.00	Secondary education teachers	113 (0.7%)	35 (0.3%)	**0.62 (0.52**–**0.76)**	**0.63 (0.52**–**0.76)**
	1-32.15	Languages and literature teacher (second level)	90 (0.6%)	20 (0.1%)	**0.62 (0.52**–**0.75)**	**0.63 (0.52**–**0.77)**
	1-32.20	Mathematics teacher (second level)	49 (0.3%)	9 (0.1%)	**0.62 (0.51**–**0.75)**	**0.62 (0.52**–**0.76)**
	1-32.30	Natural science teachers (second level)	56 (0.3%)	9 (0.1%)	**0.62 (0.51**–**0.75)**	**0.62 (0.52**–**0.76)**
	1-32.40	Social studies teacher (second level)	39 (0.2%)	9 (0.1%)	**0.62 (0.51**–**0.75)**	**0.62 (0.51**–**0.76)**
	1-32.50	Fine arts teacher (second level)	18 (0.1%)	9 (0.1%)	**0.62 (0.52**–**0.76)**	**0.63 (0.52**–**0.76)**
	1-32.60	Commercial and secretarial teacher (second level)	7 (0.0%)	4 (0.0%)	**0.63 (0.52**–**0.76)**	**0.63 (0.52**–**0.77)**
	1-32.70	Agricultural science teacher (second level)	14 (0.1%)	3 (0.0%)	**0.62 (0.51**–**0.76)**	**0.63 (0.52**–**0.76)**
	1-32.75	Domestic science and general nursing teacher (second level)	5 (0.0%)	1 (0.0%)	**0.62 (0.51**–**0.76)**	**0.62 (0.52**–**0.76)**
	1-32.80	Technical education teacher (second level)	119 (0.7%)	62 (0.5%)	**0.63 (0.52**–**0.76)**	**0.63 (0.53**–**0.77)**
	1-32.90	Other secondary education teachers	97 (0.6%)	24 (0.2%)	**0.62 (0.51**–**0.75)**	**0.62 (0.52**–**0.75)**
2-12	2-12.10	Production manager (except farm)	358 (2.2%)	182 (1.4%)	**0.75 (0.61**–**0.97)**	**0.77 (0.61**–**0.99)**
3-10	3-10.10	Government executive official	717 (4.6%)	431 (3.3%)	**0.76 (0.66**–**0.88)**	**0.77 (0.66**–**0.90)**
4-31	4-31.30	Technical service adviser	107 (0.7%)	41 (0.3%)	0.78 (0.44–1.02)	0.80 (0.45–1.02)
7-11	7-11.00	Miners and quarrymen	2 (0.0%)	6 (0.0%)	1.23 (1.00–1.47)	1.16 (0.99–1.41)
	7-11.05	Miner (general)	274 (1.7%)	454 (3.5%)	**1.24 (1.04**–**1.45)**	**1.16 (1.00**–**1.38)**
	7-11.10	Quarryman (general)	34 (0.2%)	65 (0.5%)	**1.22 (1.00**–**1.46)**	1.15 (0.99–1.40)
	7-11.20	Cutting-machine operator (mine)	3 (0.0%)	5 (0.0%)	1.21 (1.00–1.45)	1.14 (0.99–1.38)
	7-11.30	Drilling-machine operator (mine and quarry)	24 (0.1%)	27 (0.2%)	1.21 (1.00–1.44)	1.15 (0.99–1.38)
	7-11.40	Continuous-mining-machine operator	6 (0.0%)	20 (0.1%)	1.23 (1.00–1.48)	1.17 (0.99–1.42)
	7-11.50	Shot-firer (mine and quarry)	18 (0.1%)	26 (0.2%)	1.21 (1.00–1.44)	1.14 (0.99–1.38)
	7-11.70	Sampler (mine)	1 (0.0%)	1 (0.0%)	1.21 (1.00–1.45)	1.14 (0.98–1.38)
	7-11.90	Other miners and quarrymen	17 (0.1%)	24 (0.2%)	1.20 (0.99–1.44)	1.14 (0.98–1.37)
8-32	8-32.90	Other toolmakers, metal pattern makers and metal markers	9 (0.1%)	29 (0.2%)	1.26 (0.92–4.21)	1.19 (0.90–3.59)
9-51	9-51.00	Bricklayers, stonemasons and tile setters	24 (0.1%)	19 (0.1%)	**1.33 (1.15**–**1.53)**	**1.28 (1.09**–**1.49)**
	9-51.20	Bricklayer (construction)	412 (2.6%)	618 (4.8%)	**1.32 (1.17**–**1.48)**	**1.28 (1.11**–**1.45)**
	9-51.25	Bricklayer (chimney building)	3 (0.0%)	3 (0.0%)	**1.31 (1.13**–**1.51)**	**1.27 (1.07**–**1.48)**
	9-51.30	Firebrick layer	32 (0.2%)	44 (0.3%)	**1.31 (1.13**–**1.50)**	**1.27 (1.07**–**1.46)**
	9-51.40	Stonemason	8 (0.0%)	21 (0.2%)	**1.33 (1.15**–**1.54)**	**1.29 (1.09**–**1.49)**
	9-51.45	Marble setter	2 (0.0%)	6 (0.0%)	**1.32 (1.14**–**1.52)**	**1.28 (1.08**–**1.48)**
	9-51.50	Tile setter	43 (0.3%)	63 (0.5%)	**1.32 (1.14**–**1.50)**	**1.27 (1.08**–**1.46)**
	9-51.60	Paviour	9 (0.1%)	29 (0.2%)	**1.36 (1.16**–**1.63)**	**1.32 (1.10**–**1.60)**
	9-51.90	Other bricklayers–stonemasons and tile setters	29 (0.2%)	52 (0.4%)	**1.34 (1.16**–**1.55)**	**1.29 (1.10**–**1.50)**
9-89	9-89.00	Transport equipment operators nec	5 (0.0%)	11 (0.1%)	1.47 (0.96–8.47)	1.40 (0.96–7.66)

a The jobs listed in this table were associated with an increased or decreased OR of at least 20% in horseshoe penalized regression models. Bold job codes were significant in 90% credible interval (CrI). The base model used all five-digit ISCO-68 jobs that ever employed by men (*n* = 1356) and adjusted for the following covariates: age group (<45, 45–49, 50–54, 55–59, 60–64, 65–69, 70–74, >74 years), study centre, smoking (log(cigarette pack-years + 1)), smoking cessation (current smokers; >0–7; 8–15; 16–25; >25 years; never smokers). Full model further adjusted for individual cumulative exposure levels for the following occupational carcinogens: asbestos, diesel engine exhaust, hexavalent chromium, nickel, polycyclic aromatic hydrocarbons, and respirable crystalline silica.

**Table 4:** Occupations ever employed by women selected using Bayesian hierarchical penalization models (*N* = 7607).^a^

3-digit ISCO	5-digit ISCO	Job titles	Controls n (%)	Cases n (%)	ORs (90%CrIs)	
					Base model	Full model
132	1-32.00	secondary education teachers	33 (0.8%)	12 (0.4%)	**0.56 (0.40**–**0.89)**	**0.56 (0.40**–**0.89)**
	1-32.15	languages and literature teacher (second level)	57 (1.3%)	16 (0.5%)	**0.55 (0.39**–**0.86)**	**0.55 (0.39**–**0.84)**
	1-32.20	mathematics teacher (second level)	12 (0.3%)	8 (0.3%)	**0.56 (0.40**–**0.92)**	**0.56 (0.40**–**0.92)**
	1-32.30	natural science teachers (second level)	13 (0.3%)	4 (0.1%)	**0.56 (0.40**–**0.91)**	**0.56 (0.40**–**0.90)**
	1-32.40	social studies teacher (second level)	6 (0.1%)	4 (0.1%)	**0.56 (0.40**–**0.92)**	**0.56 (0.40**–**0.91)**
	1-32.50	fine arts teacher (second level)	10 (0.2%)	2 (0.1%)	**0.56 (0.40**–**0.91)**	**0.56 (0.39**–**0.89)**
	1-32.60	commercial and secretarial teacher (second level)	4 (0.1%)	1 (0.0%)	**0.56 (0.40**–**0.92)**	**0.56 (0.40**–**0.90)**
	1-32.75	domestic science and general nursing teacher (second level)	6 (0.1%)	3 (0.1%)	**0.56 (0.40**–**0.92)**	**0.56 (0.40**–**0.90)**
	1-32.80	technical education teacher (second level)	12 (0.3%)	7 (0.2%)	**0.57 (0.40**–**0.94)**	**0.57 (0.40**–**0.93)**
	1-32.90	other secondary education teachers	31 (0.7%)	11 (0.3%)	**0.56 (0.40**–**0.88)**	**0.56 (0.39**–**0.87)**
300	3-00.10	clerical supervisor (general)	57 (1.3%)	21 (0.7%)	0.80 (0.42–1.03)	0.79 (0.41–1.03)
310	3-10.10	government executive official	206 (4.9%)	140 (4.6%)	**0.71 (0.51**–**1.00)**	**0.70 (0.51**–**1.00)**

a The jobs listed in this table were associated with an increased or decreased OR of at least 20% in horseshoe penalized regression models. Bold job codes were significant in 90% credible interval (CrI). The base model used all five-digit ISCO-68 jobs that ever employed by women (*n* = 586) and adjusted for the following covariates: age group (<45, 45–49, 50–54, 55–59, 60–64, 65–69, 70–74, >74 years), study centre, smoking (log(cigarette pack-years + 1)), smoking cessation (current smokers; >0–7; 8–15; 16–25; >25 years; never smokers). Full model further adjusted for individual cumulative exposure levels for the following occupational carcinogens: asbestos, diesel engine exhaust, hexavalent chromium, nickel, polycyclic aromatic hydrocarbons, and respirable crystalline silica.

Lung cancer ORs for overlapping occupations selected by both the LASSO and Bayesian hierarchical models are shown in Table 5 for both men and women. *E*-values ranged from 2.12 to 4.85 for negative ORs and 1.88 to 1.92 for positive ORs.

**Table 5:** The risks for lung cancer of ever employed in occupations selected by both models and the related *E*-values.^a^

ISCO-68	Job title	Controls, *n* (%)	Cases, *n* (%)	ORs (95% CIs)	*E*-values for ORs
Men (*n* = 29 997)					
1-32.90	Other secondary education teachers	97 (0.6%)	24 (0.2%)	0.37 (0.22–0.60)	4.85
2-12.10	Production manager (except farm)	358 (2.2%)	182 (1.4%)	0.66 (0.54–0.81)	2.40
3-10.10	Government executive official	717 (4.6%)	431 (3.3%)	0.72 (0.62–0.83)	2.12
7-11.05	Miner (general)	274 (1.7%)	454 (3.5%)	1.28 (1.06–1.54)	1.88
9-51.20	Bricklayer (construction)	412 (2.6%)	618 (4.8%)	1.30 (1.12–1.52)	1.92
Women (*n* = 7607)					
1-32.15	Languages and literature teacher (second level)	57 (1.3%)	16 (0.5%)	0.39 (0.20–0.72)	4.57
3-10.10	Government executive official	57 (1.3%)	21 (0.7%)	0.62 (0.48–0.81)	2.61

a Lung cancer ORs and 95% confidence intervals (CIs) for selected jobs were estimated using an unconditional logistic regression model with adjustment of age group (<45, 45–49, 50–54, 55–59, 60–64, 65–69, 70–74, >74 years), study centre, smoking (log(cigarette pack-years + 1)), smoking cessation (current smokers; >0–7; 8–15; 16–25; >25 years; never smokers), as well as six individual quantitative cumulative exposure variables for the following occupational carcinogens: asbestos, diesel engine exhaust, hexavalent chromium, nickel, polycyclic aromatic hydrocarbons, and respirable crystalline silica, and ever employed by other listed jobs (ever; never). *E*-values were calculated based on ORs.

The directionalities of associations between the selected occupations and lung cancer risk largely persisted regardless of smoking status (Table 6), except for men miners who never smoked, for which the sample size was small (*n* = 64), and the estimates were imprecise. Further adjustments of the cumulative benzene exposure did not materially change the results and increased some of the observed risks (Supplementary Table S1). Sensitivity analyses restricted to jobs that were ever employed for more than 5 years in both approaches showed only minor differences (Supplementary Tables S2–S5).

**Table 6:** Subgroup analysis of associations between lung cancer and selected occupations according to smoking status.^a^

ISCO-68	Job title	Never smokers		Former smokers		Current smoker		*P*-values for multiplicative interactions^b^
		Controls, cases, *n* (%)	OR (95%CI)	Controls, cases, *n* (%)	OR (95%CI)	Controls, cases, *n* (%)	OR (95%CI)	
Men (*n* = 29 997)								
1-32.90	Other secondary education teachers	39 (0.9%), 0 (0.0%)	^c^	14 (0.3%), 15 (0.2%)	0.27 (0.06–0.99)	11 (0.6%), 3 (0.3%)	0.54 (0.25–1.18)	0.901
2-12.10	Production manager (except farm)	93 (2.1%), 6 (1.2%)	0.58 (0.23–1.25)	93 (2.0%), 113 (1.4%)	0.76 (0.41–1.36)	46 (2.6%), 18 (1.5%)	0.71 (0.53–0.95)	0.791
3-10.10	Government executive official	193 (4.6%), 13 (2.7%)	0.57 (0.30–0.99)	151 (3.3%), 246 (3.0%)	0.56 (0.37–0.85)	104 (6.1%), 41 (3.5%)	0.81 (0.65–1.01)	0.167
7-11.05	Miner (general)	57 (1.3%), 7 (1.5%)	0.72 (0.27–1.64)	79 (1.7%), 268 (3.3%)	1.61 (0.94–2.77)	31 (1.7%), 45 (3.9%)	1.17 (0.89–1.56)	0.107
9-51.20	Bricklayer (construction)	90 (2.1%), 15 (3.2%)	1.17 (0.62–2.08)	120 (2.6%), 393 (5.0%)	1.13 (0.72–1.77)	55 (3.1%), 54 (4.7%)	1.47 (1.17–1.86)	0.599
Women (*n* = 7607)								
1-32.15	Languages and literature teacher (second level)	29 (1.1%), 5 (0.6%)	0.63 (0.21–1.53)	10 (1.1%), 9 (0.5%)	0.54 (0.06–3.66)	5 (2.1%), 2 (1.2%)	0.53 (0.19–1.45)	0.326
3-10.10	Government executive official	114 (4.5%), 34 (4.1%)	0.68 (0.44–1.01)	45 (5.3%), 86 (5.3%)	0.41 (0.10–1.53)	17 (7.4%), 5 (3.1%)	0.65 (0.42–1.03)	0.685

a Unconditional logistic regression models were used for the assessment of the association with risk of lung cancer. The ORs and 95% CIs for job-specific risk were calculated, adjusting for age group (<45, 45–49, 50–54, 55–59, 60–64, 65–69, 70–74, >74 years), study centre, as well as six individual quantitative cumulative exposure variables for the following occupational carcinogens: asbestos, diesel engine exhaust, hexavalent chromium, nickel, polycyclic aromatic hydrocarbons, and respirable crystalline silica, and ever employed by other selected jobs (ever; never). For former smokers, smoking cessation (current smokers; >0–7; 8–15; 16–25; >25 years; never smokers), smoking (log(cigarette pack-years + 1)) were further adjusted and for current smokers, smoking (log(cigarette pack-years + 1)) were further adjusted.

b *P*-values were the joint effect of ever-employed in the respective job (never; ever) and ever-smoking (never; ever).

c Men subjects ever employed as other secondary education teachers were all controls.

## Discussion

We implemented two data-driven statistical approaches to agnostically explore occupations associated with lung cancer while accounting for smoking and occupational exposures to multiple known lung carcinogens.

No established method exists for occupation- or exposure-specific agnostic analyses in occupational epidemiology; we therefore used two distinct modelling approaches. By assumptions, the LASSO approach selected individual occupations without clear clustering of similar ISCO-68 occupations. In contrast, the Bayesian approach incorporated the hierarchical nature of the ISCO-68 classification system, where similar five-digit occupations are organized within three-digit unit groups. Assuming five-digit occupations within a three-digit group share exposure- and disease-related characteristics, this approach estimated five-digit effects using information at both levels. As a result, the Bayesian approach selected clustered occupations within the same three-digit groups. Furthermore, the Bayesian approach enabled direct comparisons of continuous effect sizes and uncertainties across different occupations, allowing for flexible screening and selection criteria based on both parameters. Although the two modelling approaches relied on different assumptions about how disease risks differ in neighbouring five-digit occupations, there were overlaps in selected occupations. While our current analyses are focused on higher specificity, both the LASSO and Bayesian approaches may be adjusted to allow for greater sensitivity for more explorative screening of occupations related to disease.

Construction bricklayers and miners were selected by both modelling approaches with an elevated risk of lung cancer for men, while accounting for smoking and exposure to six known lung carcinogens and benzene. Increased risks for both occupations have been identified previously in other studies as well as in the SYNERGY population [17–20]. In addition to confirming previous hypothesis-driven research, our analyses further showed that the elevated risk is not fully explained by known occupational risk factors.

This residual effect may be driven by several factors. First, there may be interactive effects between known exposures and/or other covariates that were unaccounted for. Asbestos, crystalline silica, diesel engine exhaust and hexavalent chromium are known carcinogens associated with occupations such as bricklayer and miner. However, pairwise interaction effects between occupational lung carcinogen exposures have been previously reported in the SYNERGY population as either small or null, including those for asbestos, crystalline silica, and hexavalent chromium [5]. Pairwise interactive effects between individual lung carcinogen exposure and cigarette smoking have been reported as small but positive on the additive scale; this may partially explain the residual lung cancer risks observed [7, 21, 22]. However, our stratified analysis by smoking status showed positive central estimates between the bricklayer occupation and lung cancer, regardless of smoking status. Second, the observed effect may be caused by inaccurate exposure estimates for known exposures in bricklayers and miners. However, it is unlikely that only exposures for bricklayers and miners were inaccurate in our job-exposure matrix, while exposures for other jobs remain relatively accurate. Third, there may be additional risk factors that were not considered. Regarding this third explanation, the *E*-values were 1.88 and 1.92 for construction bricklayers and miners, respectively. To explain the observed effect in miners, for instance, the unaccounted factor must be associated with lung cancer with an OR of 1.88 and be 1.88 times more likely to be present among miners than nonminers. For underground miners, radon exposure may explain some of the observed positive effects [23, 24]. Future research may investigate exposures for bricklayers to elucidate if they involve unknown risk factors associated with lung cancer.

We also found that ever working as a secondary teacher was negatively associated with lung cancer risk for both men and women. Lower lung cancer risks for ‘teachers’ have also been identified previously [25–27]. Several other teaching-related occupations were also selected. The *E*-values of the estimates for these occupations ranged from 4.57 to 4.85, suggesting that unmeasured factors must have a strong relation with both ever employment in teaching-related occupations and lung cancer risk to explain the observed decreased risks. Residual confounding from smoking is unlikely to be the main driver of these observed associations, as stratified analyses by smoking status showed similar central estimates. These negative associations may reflect reduced exposure to known carcinogens or unknown protective factors; clarifying them could inform lung cancer prevention. Several other jobs were also selected to have negative associations with lung cancer risks, including mechanical engineers and production managers for men, and government executive officials for both sexes. *E*-values for these estimated associations exceeded 2.0, suggesting that the observed associations are reasonably robust, and the possibility of confounding by unmeasured factors appears to be low.

The LASSO models selected a few notable occupations positively associated with lung cancer. For men, occupations with the highest association magnitude with lung cancer in the LASSO full model but were not identified in the Bayesian model include dockers, excavating machine operators, carpenters, painters, and welders. Various studies have shown that these occupations are likely to be associated with lung cancer [28–32], including previous exploratory analyses based on a subset of our current study population [33, 34]. For women, other production workers, waiters, and charworkers were identified as having relatively strong associations with lung cancer. These findings are also in accordance with earlier studies, which identified professional cleaning, restaurant work, and manufacturing assembly as risk factors [35, 36].

Our study has several advantages. We combined two modelling approaches to agnostically explore occupation-level factors without a hypothesis related to a specific exposure or occupation. In addition to identifying high-risk occupations for lung cancer, our approach also allowed for the identification of occupations with lower lung cancer risks. Negative associations and protective factors receive less attention than risk factors in hypothesis-driven research, yet they may inform aetiology and interventions [37]. Furthermore, our data-driven approaches were applied to a large, population-based pooled case–control study with comprehensive covariate information, including full occupational history and quantitative assessments of occupational exposure to six known lung carcinogens as well as cigarette smoking and cessation. Lastly, almost all highlighted high-risk occupations are well established in the literature, suggesting that our methods have sufficient sensitivity and specificity in this dataset.

There are also some limitations in our study. First, our main models considered only ever-employment without accounting for additional aspects such as employment duration and timing of employment. However, sensitivity analyses restricted to jobs held for 5 years or more by subjects did not change our main results. Another limitation is the lack of information on second-hand smoke exposure, other nonoccupational exposures, and comorbidities, which could not be accounted for in the models and may have contributed to residual confounding across occupations. Third, our final inference model to calculate occupation-specific associations may produce estimates with larger effect sizes and smaller uncertainties, as occupation variable selection was performed with separate models. However, boosting implemented in the LASSO models may mitigate this effect, and our inference model ORs and uncertainties were mostly similar to those estimated in the Bayesian hierarchical model, which suggests limited bias in effect sizes and uncertainties.

## Conclusion

Using two agnostic, data-driven approaches conditioned on known lung cancer risk factors, we identified several occupations positively and negatively associated with lung cancer in a large-scale, pooled case–control study, demonstrating that such methods can screen for occupational risks that may be missed by hypothesis-driven designs. In our population, the associations observed in both directions were not fully explained by the exposures and factors investigated. For positively associated occupations, such as bricklayers, stonemasons and tile setters and miners, the residual risk is consistent with a possible genuine excess and identifies these groups as priorities for targeted exposure assessment to elucidate whether the excess is driven by exposure misclassification or unrecognized occupational risk factors. The negative associations, for example, among teachers, similarly warrant follow-up to identify the protective factors, whether occupational or lifestyle-related, that underlie the reduced risk. As smoking prevalence declines, occupational information may add increased value to lung cancer risk prediction models; for that purpose, it should be based on established causal agents, such as asbestos, crystalline silica, and diesel engine exhaust, and not on exploratory associations such as ours, which are intended to flag occupations for further study rather than to inform screening directly.

## Ethics approval

Ethical approvals for the SYNERGY project were obtained according to the legislation in each participating country and the IARC institutional review board.

## Supplementary Material

dyag183_Supplementary_Data

## Data Availability

The data are not publicly available.
