# Supplementary material for: Data-driven exploration of occupations and lung cancer risks using regularized regression models
Source: Int J Epidemiol. 2026 Oct 23;55(6):dyag183. doi: 10.1093/ije/dyag183 (PMC13692792; doi:10.1093/ije/dyag183)
Supplement: Supplementary file 1 [file dyag183_supplementary_data.zip › ije-2025-08-1518-File003.docx]

# Supplementary materials for “Data-driven exploration of occupations and lung cancer risks using regularised regression models”

**Supplementary table 1. Sensitivity analysis of associations between lung cancer and the selected jobs with further adjustment of cumulative exposure to benzene.**

**Supplementary table 2. Sensitivity analysis of occupations held more than 5 years selected using Bayesian hierarchical penalization models in men (n=29944).**

**Supplementary table 3. Sensitivity analysis of occupations held more than 5 years selected using Bayesian hierarchical penalization models in women (n=7579).**

**Supplementary table 4. Sensitivity analysis of occupations held more than 5 years selected using least absolute shrinkage and selection operator (LASSO) penalized regression models in men (n=29944).**

**Supplementary table 5. Sensitivity analysis of occupations held more than 5 years selected using least absolute shrinkage and selection operator (LASSO) penalized regression models in women (n=7579).**

## Supplementary table 1. Sensitivity analysis of associations between lung cancer and the selected jobs with further adjustment of cumulative exposure to benzene.

| **ISCO-68** | **Job title** | **Controls**  **n (%)** | **Cases**  **n (%)** | **ORs (95%CI)** |
| --- | --- | --- | --- | --- |
| Men (n=21,750) |  |  |  |  |
| 1-32.90 | other secondary education teachers | 73 (0.6%) | 19 (0.2%) | 0.34 (0.20, 0.55) |
| 2-12.10 | production manager (except farm) | 263 (2.2%) | 118 (1.2%) | 0.61 (0.49, 0.76) |
| 3-10.10 | government executive official | 441 (3.8%) | 236 (2.5%) | 0.68 (0.58, 0.81) |
| 7-11.05 | miner (general) | 162 (1.4%) | 254 (2.7%) | 1.36 (1.09, 1.69) |
| 9-51.20 | bricklayer (construction) | 307 (2.6%) | 466 (5.0%) | 1.57 (1.34, 1.84) |
| Women (n=6,299) |  |  |  |  |
| 1-32.15 | languages and literature teacher (second level) | 57 (1.3%) | 16 (0.5%) | 0.35 (0.19, 0.61) |
| 3-10.10 | government executive official | 206 (4.9%) | 140 (4.6%) | 0.76 (0.57, 1.01) |

Notes: The jobs presented here were jobs selected by both Bayesian hierarchical and least absolute shrinkage and selection operator (LASSO) approaches (per family error rate [PFER] =10). Unconditional logistic regression models were used for the assessment of the association with risk of lung cancer. The ORs and 95% CIs for 5-digit job-specific risk were calculated, adjusting for age group (<45; 45–49; 50–54; 55–59; 60–64; 65–69; 70–74; >74 years), study center, smoking (log(cigarette pack-years+1)), smoking cessation (current smokers; >0-7; 8-15; 16-25; >25 years; never smokers), as well as six individual quantitative cumulative exposure variables for the following occupational carcinogens: asbestos, diesel engine exhaust, hexavalent chromium, nickel, polycyclic aromatic hydrocarbons, and respirable crystalline silica and benzene, and ever employed by other selected jobs (ever; never).

## Supplementary table 2. Sensitivity analysis of occupations held more than 5 years selected using Bayesian hierarchical penalization models in men (n=29 944).

| **3-digit ISCO** | **5-digit ISCO** | **Job title** | **Controls**  **n (%)** | **Cases**  **n (%)** | **OR (90%CrI)** |
| --- | --- | --- | --- | --- | --- |
| 0-24 | 0-24.10 | mechanical engineer (general) | 165 (1.0%) | 50 (0.4%) | **0.55 (0.37, 0.98)** |
| 0-83 | 0-83.00 | systems analysts | 11 (0.1%) | 1 (0.0%) | 0.58 (0.23, 1.03) |
|  | 0-83.10 | systems analyst | 63 (0.4%) | 11 (0.1%) | 0.55 (0.23, 1.02) |
| 1-31 | 1-31.55 | teacher in education (third level) | 8 (0.0%) | 7 (0.1%) | 2.58 (0.80, 18.36) |
| 1-32 | 1-32.00 | secondary education teachers | 113 (0.7%) | 35 (0.3%) | **0.63 (0.53, 0.77)** |
|  | 1-32.15 | languages and literature teacher (second level) | 90 (0.6%) | 20 (0.1%) | **0.63 (0.52, 0.76)** |
|  | 1-32.20 | mathematics teacher (second level) | 49 (0.3%) | 9 (0.1%) | **0.62 (0.52, 0.76)** |
|  | 1-32.30 | natural science teachers (second level) | 56 (0.3%) | 9 (0.1%) | **0.63 (0.52, 0.76)** |
|  | 1-32.40 | social studies teacher (second level) | 39 (0.2%) | 9 (0.1%) | **0.62 (0.52, 0.76)** |
|  | 1-32.50 | fine arts teacher (second level) | 18 (0.1%) | 9 (0.1%) | **0.63 (0.52, 0.77)** |
|  | 1-32.60 | commercial and secretarial teacher (second level) | 7 (0.0%) | 4 (0.0%) | **0.63 (0.53, 0.78)** |
|  | 1-32.70 | agricultural science teacher (second level) | 14 (0.1%) | 3 (0.0%) | **0.63 (0.52, 0.76)** |
|  | 1-32.75 | domestic science and general nursing teacher (second level) | 5 (0.0%) | 1 (0.0%) | **0.63 (0.52, 0.76)** |
|  | 1-32.80 | technical education teacher (second level) | 119 (0.7%) | 62 (0.5%) | **0.63 (0.53, 0.77)** |
|  | 1-32.90 | other secondary education teachers | 97 (0.6%) | 24 (0.2%) | **0.62 (0.52, 0.76)** |
| 2-12 | 2-12.10 | production manager (except farm) | 358 (2.2%) | 182 (1.4%) | **0.76 (0.61, 0.98)** |
| 3-10 | 3-10.10 | government executive official | 717 (4.6%) | 431 (3.3%) | **0.77 (0.66, 0.89)** |
| 4-31 | 4-31.30 | technical service adviser | 107 (0.7%) | 41 (0.3%) | 0.79 (0.44, 1.02) |
| 9-51 | 9-51.00 | bricklayers, stonemasons and tile setters | 24 (0.1%) | 19 (0.1%) | **1.29 (1.10, 1.50)** |
|  | 9-51.20 | bricklayer (construction) | 412 (2.6%) | 618 (4.8%) | **1.28 (1.12, 1.46)** |
|  | 9-51.25 | bricklayer (chimney building) | 3 (0.0%) | 3 (0.0%) | **1.27 (1.08, 1.48)** |
|  | 9-51.30 | firebrick layer | 32 (0.2%) | 44 (0.3%) | **1.27 (1.08, 1.47)** |
|  | 9-51.40 | stonemason | 8 (0.0%) | 21 (0.2%) | **1.29 (1.10, 1.50)** |
|  | 9-51.45 | marble setter | 2 (0.0%) | 6 (0.0%) | **1.28 (1.10, 1.49)** |
|  | 9-51.50 | tile setter | 43 (0.3%) | 63 (0.5%) | **1.27 (1.09, 1.47)** |
|  | 9-51.60 | paviour | 9 (0.1%) | 29 (0.2%) | **1.32 (1.11, 1.59)** |
|  | 9-51.90 | other bricklayers, stonemasons and tile setters | 29 (0.2%) | 52 (0.4%) | **1.30 (1.11, 1.51)** |
| 9-89 | 9-89.00 | transport equipment operators nec | 5 (0.0%) | 11 (0.1%) | 1.49 (0.96, 8.49) |

Notes: The jobs listed in this table were associated with an increased or decreased odds ratio (OR) of at least 20% in horseshoe penalized regression models. Bold job codes were also significant in 90% credible interval (CrI). Jobs within the same white or grey backgrounds were from the same 3-digit job unit level.

Model used all 5-digit ISCO-68 jobs (n=1355) that ever employed by men subjects for more than 5 years and adjusted for the following covariates: age group (<45; 45–49; 50–54; 55–59; 60–64; 65–69; 70–74; >74 years), study center, smoking (log(cigarette pack-years+1)), smoking cessation (current smokers; >0-7; 8-15; 16-25; >25 years; never smokers), individual cumulative exposure levels for the following occupational carcinogens: asbestos, diesel engine exhaust, hexavalent chromium, nickel, polycyclic aromatic hydrocarbons, and respirable crystalline silica.

## Supplementary table 3. Sensitivity analysis of occupations held more than 5 years selected using Bayesian hierarchical penalization models in women (n=7579).

| **3-digit ISCO** | **5-digit ISCO** | **Job title** | **Controls**  **n (%)** | **Cases**  **n (%)** | **OR (90%CrI)** |
| --- | --- | --- | --- | --- | --- |
| 1-32 | 1-32.00 | secondary education teachers | 33 (0.8%) | 12 (0.4%) | **0.56 (0.39, 0.92)** |
|  | 1-32.15 | languages and literature teacher (second level) | 57 (1.3%) | 16 (0.5%) | **0.54 (0.38, 0.81)** |
|  | 1-32.20 | mathematics teacher (second level) | 12 (0.3%) | 8 (0.3%) | **0.56 (0.39, 0.94)** |
|  | 1-32.30 | natural science teachers (second level) | 13 (0.3%) | 4 (0.1%) | **0.56 (0.39, 0.92)** |
|  | 1-32.40 | social studies teacher (second level) | 6 (0.1%) | 4 (0.1%) | **0.56 (0.39, 0.93)** |
|  | 1-32.50 | fine arts teacher (second level) | 10 (0.2%) | 2 (0.1%) | **0.55 (0.39, 0.92)** |
|  | 1-32.60 | commercial and secretarial teacher (second level) | 4 (0.1%) | 1 (0.0%) | **0.56 (0.39, 0.93)** |
|  | 1-32.75 | domestic science and general nursing teacher (second level) | 6 (0.1%) | 3 (0.1%) | **0.56 (0.39, 0.93)** |
|  | 1-32.80 | technical education teacher (second level) | 12 (0.3%) | 7 (0.2%) | **0.56 (0.39, 0.95)** |
|  | 1-32.90 | other secondary education teachers | 31 (0.7%) | 11 (0.3%) | **0.55 (0.39, 0.89)** |
| 3-00 | 3-00.10 | clerical supervisor (general) | 57 (1.3%) | 21 (0.7%) | 0.80 (0.42, 1.03) |
| 3-10 | 3-10.10 | government executive official | 206 (4.9%) | 140 (4.6%) | **0.70 (0.51, 1.00)** |

Notes: The jobs listed in this table were associated with an increased or decreased odds ratio (OR) of at least 20% in horseshoe penalized regression models. Bold job codes were also significant in 90% credible interval (CrI). Jobs within the same white or grey backgrounds were from the same 3-digit job unit level.

Model used all 5-digit ISCO-68 jobs (n=584) that ever employed by women subjects for more than 5 years and adjusted for the following covariates: age group (<45; 45–49; 50–54; 55–59; 60–64; 65–69; 70–74; >74 years), study center, smoking (log(cigarette pack-years+1)), smoking cessation (current smokers; >0-7; 8-15; 16-25; >25 years; never smokers), individual cumulative exposure levels for the following occupational carcinogens: asbestos, diesel engine exhaust, hexavalent chromium, nickel, polycyclic aromatic hydrocarbons, and respirable crystalline silica.

## Supplementary table 4. Sensitivity analysis of occupations held more than 5 years selected using least absolute shrinkage and selection operator (LASSO) penalized regression models in men (n=29 944).

| **ISCO-68** | **Job title** | **Controls**  **n (%)** | **Cases**  **n (%)** | **Lung cancer association directionality ^a^** |
| --- | --- | --- | --- | --- |
| 0-24.10 | mechanical engineer (general) | 165 (1.0%) | 50 (0.4%) | -0.582 |
| 1-32.90 | other secondary education teachers | 97 (0.6%) | 24 (0.2%) | -0.580 |
| 1-33.20 | first level education teachers | 150 (0.9%) | 53 (0.4%) | -0.292 |
| 2-12.10 | production manager (except farm) | 358 (2.2%) | 182 (1.4%) | -0.350 |
| 2-19.40 | administration manager | 188 (1.2%) | 81 (0.6%) | -0.207 |
| 3-10.10 | government executive official | 717 (4.6%) | 431 (3.3%) | -0.273 |
| 3-31.10 | bookkeeper (general) | 211 (1.3%) | 102 (0.8%) | -0.278 |
| 3-93.10 | office clerk (general) | 824 (5.3%) | 508 (3.9%) | -0.152 |
| 4-31.30 | technical service adviser | 107 (0.7%) | 41 (0.3%) | -0.490 |
| 4-32.20 | commercial traveller | 483 (3.0%) | 345 (2.6%) | -0.187 |
|  | armed forces ^b^ | 834 (5.4%) | 889 (7.0%) | +0.102 |
| 7-11.05 | miner (general) | 274 (1.7%) | 454 (3.5%) | +0.202 |
| 8-34.20 | lathe operator | 356 (2.2%) | 307 (2.3%) | -0.148 |
| 8-49.10 | machinery mechanic (general) | 312 (1.9%) | 388 (2.9%) | +0.202 |
| 8-72.10 | gas and electric welder (general) | 241 (1.5%) | 340 (2.6%) | +0.186 |
| 9-31.20 | building painter | 265 (1.6%) | 351 (2.7%) | +0.250 |
| 9-51.20 | bricklayer (construction) | 412 (2.6%) | 618 (4.8%) | +0.229 |
| 9-54.10 | carpenter (general) | 107 (0.7%) | 171 (1.3%) | +0.460 |
| 9-71.20 | docker | 71 (0.4%) | 109 (0.8%) | +0.403 |
| 9-74.20 | excavating machine operator | 75 (0.5%) | 119 (0.9%) | +0.356 |
| 9-85.60 | lorry and van driver (long-distance transport) | 478 (3.0%) | 547 (4.2%) | +0.100 |
| 9-99.10 | labourer | 1200 (7.9%) | 1416 (11.7%) | +0.087 |

^a^ Selected jobs indicated by “+” or “-” and the shrank coefficient per job title, representing “positive” or “negative” associations with lung cancer risk.

^b^ No ISCO-68 code is available for armed forces.

Notes: Least absolute shrinkage and selection operator (LASSO) regression with stability selection was set at a cut-off rate of 0.75 (which means any job with a selection probability higher than 0.75 will be selected) and per family error rate (PFER) of 10 (maximum tolerated number of Type I errors among selections was 10).

Model used all 5-digit ISCO-68 jobs (n=1355) that ever employed by men subjects for more than 5 years and adjusted for the following covariates: age group (<45; 45–49; 50–54; 55–59; 60–64; 65–69; 70–74; >74 years), study center, smoking (log(cigarette pack-years+1)), smoking cessation (current smokers; >0-7; 8-15; 16-25; >25 years; never smokers), individual cumulative exposure levels for the following occupational carcinogens: asbestos, diesel engine exhaust, hexavalent chromium, nickel, polycyclic aromatic hydrocarbons, and respirable crystalline silica.

## Supplementary table 5. Sensitivity analysis of occupations held more than 5 years selected using least absolute shrinkage and selection operator (LASSO) penalized regression models in women (n=7579).

| **ISCO-68** | **Job title** | **Controls**  **n (%)** | **Cases**  **n (%)** | **Lung cancer association directionality ^a^** |
| --- | --- | --- | --- | --- |
| 1-32.15 | languages and literature teacher (second level) | 57 (1.3%) | 16 (0.5%) | -0.275 |
| 1-33.20 | first level education teachers | 156 (3.7%) | 61 (1.9%) | -0.139 |
| 3-00.10 | clerical supervisor (general) | 57 (1.3%) | 21 (0.7%) | -0.059 |
| 3-10.10 | government executive official | 206 (4.9%) | 140 (4.6%) | -0.327 |
| 3-31.10 | bookkeeper (general) | 178 (4.2%) | 110 (3.6%) | -0.036 |
| 3-39.40 | finance clerk | 50 (1.2%) | 44 (1.4%) | +0.150 |
| 3-94.10 | receptionist (general) | 95 (2.2%) | 41 (1.3%) | -0.149 |
| 5-31.30 | cook, except private service | 124 (2.9%) | 75 (2.4%) | -0.134 |
| 5-32.10 | waiter general | 151 (3.6%) | 197 (6.6%) | +0.143 |
| 5-52.20 | charworkers | 348 (8.6%) | 339 (11.9%) | +0.120 |
| 9-49.90 | other production and related workers nec | 60 (1.4%) | 76 (2.4%) | +0.336 |

^a^ Selected jobs indicated by “+” or “-” and the shrank coefficient per job title, representing “positive” or “negative” associations with lung cancer risk.

Notes: Least absolute shrinkage and selection operator (LASSO) regression with stability selection was set at a cut-off rate of 0.75 (which means any job with a selection probability higher than 0.75 will be selected) and per family error rate (PFER) of 10 (maximum tolerated number of Type I errors among selections was 10).

Model used all 5-digit ISCO-68 jobs (n=584) that ever employed by women subjects for more than 5 years and adjusted for the following covariates: age group (<45; 45–49; 50–54; 55–59; 60–64; 65–69; 70–74; >74 years), study center, smoking (log(cigarette pack-years+1)), smoking cessation (current smokers; >0-7; 8-15; 16-25; >25 years; never smokers), individual cumulative exposure levels for the following occupational carcinogens: asbestos, diesel engine exhaust, hexavalent chromium, nickel, polycyclic aromatic hydrocarbons, and respirable crystalline silica.
